# Os *Top 10* Artigos Originais Publicados nos *Arquivos Brasileiros de Cardiologia* e na *Revista Portuguesa de Cardiologia* em 2019

**DOI:** 10.36660/abc.20200176

**Published:** 2020-03-20

**Authors:** Gláucia Maria Moraes de Oliveira, Ricardo Fontes-Carvalho, Lino Gonçalves, Nuno Cardim, Carlos Eduardo Rochitte

**Affiliations:** 1 Faculdade de Medicina Universidade Federal do Rio de Janeiro Rio de JaneiroRJ Brasil Faculdade de Medicina – Universidade Federal do Rio de Janeiro , Rio de Janeiro , RJ – Brasil; 2 Instituto do Coração Edson Saad Universidade Federal do Rio de Janeiro Rio de JaneiroRJ Brasil Instituto do Coração Edson Saad – Universidade Federal do Rio de Janeiro , Rio de Janeiro , RJ – Brasil; 3 Departamento de Cardiologia Centro Hospitalar de Vila Nova de Gaia Vila Nova de Gaia Portugal Departamento de Cardiologia – Centro Hospitalar de Vila Nova de Gaia , Vila Nova de Gaia – Portugal; 4 Departamento de Cirurgia e Fisiologia Faculdade de Medicina Universidade do Porto Porto Portugal Departamento de Cirurgia e Fisiologia – Faculdade de Medicina – Universidade do Porto , Porto – Portugal; 5 Departamento de Cardiologia Centro Hospitalar Universitário de Coimbra Coimbra Portugal Departamento de Cardiologia – Centro Hospitalar e Universitário de Coimbra , Coimbra – Portugal; 6 Faculdade de Medicina Universidade de Coimbra Coimbra Portugal Faculdade de Medicina – Universidade de Coimbra , Coimbra – Portugal; 7 Departamento de Cardiologia Hospital da Luz Lisboa Portugal Departamento de Cardiologia do Hospital da Luz , Lisboa - Portugal; 8 Faculdade de Ciências Médicas Universidade Nova de Lisboa Lisboa Portugal Faculdade de Ciências Médicas da Universidade Nova de Lisboa , Lisboa - Portugal; 9 Instituto do Coração Faculdade de Medicina Universidade de São Paulo São PauloSP Brasil Instituto do Coração (InCor) – Faculdade de Medicina da Universidade de São Paulo , São Paulo , SP – Brasil; 10 Hospital do Coração São PauloSP Brasil Hospital do Coração (HCOR), São Paulo , SP – Brasil

**Keywords:** Doenças Cardiovasculares, Revistas como Assunto, Portais de Acesso à Revistas Científicas, Fator de Impacto de Revistas, Publicações Científicas e Técnicas, Publicação Periódica, Sistemas de Avaliação das Publicações

## Introdução

Há vários séculos Portugal e Brasil têm uma longa tradição de cooperação. Na Medicina, sobretudo na área de Cardiologia, existe um longo histórico de colaboração entre as duas sociedades científicas – a Sociedade Brasileira de Cardiologia e a Sociedade Portuguesa de Cardiologia – que se estende aos respetivos periódicos.

Os *Arquivos Brasileiros de Cardiologia* ( *Arq Bras Cardiol* ), publicação científica oficial da Sociedade Brasileira de Cardiologia, com fator de impacto de 1,679 em 2018 (JCR), é a revista de maior repercussão em Cardiologia no Brasil e da América Latina. Esse fato pode ser exemplificado pelo número crescente de artigos submetidos aos *Arq Bras Cardiol* (650 em 2017, 771 em 2018 e 734 em 2019) e pelo índice h5 de 31 e mediana h5 de 39, com taxa de aceitação inferior a 20%.

A Revista Portuguesa de Cardiologia ( *Rev Port Cardiol* ) tem uma publicação ininterrupta desde 1982, sendo o veículo oficial da Sociedade Portuguesa de Cardiologia, membro institucional da Sociedade Europeia de Cardiologia. É um periódico de impacto global, indexado em PubMed, Elsevier, Science Direct e SCOPUS, com fator de impacto de 0.79 no ano de 2018.

Naquele ano, pela primeira vez, as duas revistas atuaram em conjunto para publicar um resumo com os trabalhos originais mais relevantes veiculados por elas em 2018. ^1^ Face ao grande sucesso dessa iniciativa, os corpos editoriais dos dois periódicos decidiram cooperar novamente para selecionar as melhores publicações de 2019. Os trabalhos dos *Arq Bras Cardiol* listados neste artigo foram os selecionados para o Prêmio de Publicações da Sociedade Brasileira de Cardiologia. Cabe ressaltar que, em 2019, tivemos a presença de Nuno Cardim, autor do melhor estudo da *Rev Port Cardiol* em 2019, nas premiações do 74º Congresso Brasileiro de Cardiologia, em Porto Alegre. Pretendemos assim reforçar os laços culturais das principais revistas de Cardiologia em língua portuguesa, que representam o que há de melhor nas publicações voltadas para uma população crescente de cerca de 250 milhões de pessoas no mundo.

Dada a qualidade geral dos artigos publicados, esta seleção foi uma tarefa difícil, possivelmente imperfeita, mas permite destacar vários trabalhos de grande mérito na Cardiologia. Nas Tabelas 1 e 2 estão resumidos os dez melhores artigos publicados em cada revista em 2019.

**Tabela 1:** – Lista com a seleção dos dez melhores artigos publicados nos *Arquivos Brasileiros de Cardiologia* em 2019

Autor	Título e link
Faria AP et al. ^7^	Proposta de um Escore Inflamatório de Citocinas e Adipocinas Plasmáticas Associado à Hipertensão Resistente, mas Dependente dos Parâmetros de Obesidade http://www.scielo.br/scielo.php?script=sci_arttext&pid=S0066-782X2019000400383&lng=pt&nrm=iso&tlng=pt
Dippe Jr. et al. ^18^	Estudo de Perfusão Miocárdica em Obesos sem Doença Cardíaca Isquêmica Conhecida http://www.scielo.br/scielo.php?script=sci_arttext&pid=S0066-782X2019000200121&lng=en&nrm=iso&tlng=pt
Reuter CP et al. ^5^	Relação entre Dislipidemia, Fatores Culturais e Aptidão Cardiorrespiratória em Escolares http://www.scielo.br/scielo.php?script=sci_arttext&pid=S0066-782X2019000600729&lng=en&nrm=iso&tlng=pt
Villela PTM et al. ^9^	A Preferência ao Sal está Relacionada à Hipertensão e não ao Envelhecimento http://www.scielo.br/scielo.php?pid=S0066-782X2019005015104&script=sci_arttext&tlng=pt
Barros MVL et al. ^27^	Alteração Contrátil Segmentar Ventricular Esquerda é Preditor Independente de Cardiotoxicidade em Pacientes com Câncer de Mama em Tratamento Quimioterápico http://www.scielo.br/scielo.php?script=sci_arttext&pid=S0066-782X2019000100050&lng=pt&nrm=iso&tlng=pt
Kiyose AT et al. ^28^	Comparação de Próteses Biológicas e Mecânicas para Cirurgia de Válvula Cardíaca: Revisão Sistemática de Estudos Controlados Randomizados http://www.scielo.br/scielo.php?script=sci_arttext&pid=S0066-782X2019000300292&lng=en&nrm=iso&tlng=pt
Eickemberg M et al. ^6^	Indicadores de Adiposidade Abdominal e Espessura Médio-Intimal de Carótidas: Resultados do Estudo Longitudinal de Saúde do Adulto - ELSA-Brasil http://www.scielo.br/scielo.php?script=sci_arttext&pid=S0066-782X2019000300220&lng=en&nrm=iso&tlng=pt
Effting PS et al. ^8^	Exercício Resistido Modula Parâmetros de Estresse Oxidativo e Conteúdo de TNF-α no Coração de Camundongos com Obesidade Induzida por Dieta http://www.scielo.br/scielo.php?script=sci_arttext&pid=S0066-782X2019000500545&lng=es&nrm=iso&tlng=pt
Avila WS et al. ^29^	Gravidez em portadoras de cardiopatias congênitas complexas. Um constante desafio http://www.scielo.br/scielo.php?pid=S0066-782X2019005019101&script=sci_arttext&tlng=pt
Barbosa JE et al. ^17^	Perfil da Expressão do mRNA do Nrf2, NF-κB e PPARβ/δ em Pacientes com Doença Arterial Coronariana http://www.scielo.br/scielo.php?script=sci_arttext&pid=S0066-782X2019005001205&lng=en&nrm=iso&tlng=pt

**Tabela 2:** – Lista com a seleção dos dez melhores artigos publicados na *Revista Portuguesa de Cardiologia* em 2019

Autores e Referencias	Título do artigo
P Marques Silva et al. ^2^	Alterações persistentes do perfil lipídico na prática clínica nos doentes adultos portugueses com dislipidemia em tratamento com antidislipidémicos. Dados do estudo DISGEN-LIPID https://www.revportcardiol.org/pt-suboptimal-lipid-levels-in-clinical-articulo-S0870255119304998
P Marques Silva et al. ^4^	Prevalência de fatores de risco cardiovascular e outras comorbilidades em doentes com hipertensão arterial assistidos nos Cuidados de Saúde Primários: estudo PRECISE https://www.revportcardiol.org/pt-prevalencia-fatores-risco-cardiovascular-e-articulo-S0870255118302762
R Calé et al. ^12^	Tempo para a reperfusão num subgrupo de doentes de alto risco com enfarte agudo do miocárdio submetidos a angioplastia primária https://www.revportcardiol.org/pt-time-reperfusion-in-high-risk-patients-articulo-S087025511930527X
J Pinto Monteiro et al. ^13^	KAsH: Uma nova ferramenta para previsão de mortalidade hospitalar em doentes com Enfarte Agudo do Miocárdio https://www.revportcardiol.org/pt-kash-a-new-tool-predict-articulo-S0870255119305785
D Bento et al. ^16^	Prognóstico a curto e médio prazo da síndrome de Takotsubo numa população portuguesa https://www.revportcardiol.org/pt-short-medium-term-prognosis-takotsubo-syndrome-articulo-S0870255118300933
D Bonhorst et al. ^20^	Implantação de dispositivos de ressincronização e/ou desfibrilhação em doentes com insuficiência cardíaca: dados da vida real – o Estudo Síncrone https://www.revportcardiol.org/pt-implantacao-dispositivos-ressincronizacao-e-ou-desfibrilhacao-articulo-S0870255117306893
L Fernandes et al. ^19^	Acidente vascular cerebral isquémico em doentes previamente anticoagulados por fibrilhação auricular não valvular: porque acontece? https://www.revportcardiol.org/pt-acidente-vascular-cerebral-isquemico-em-articulo-S0870255118300155
C Ruivo et al. ^25^	The SHIFT model combines clinical, electrocardiographic and echocardiographic parameters to predict Sudden Cardiac Death in Hypertrophic Cardiomyopathy
A Sousa et al. ^23^	Caracterização molecular dos doentes portugueses com miocardiopatia dilatada https://www.revportcardiol.org/pt-molecular-characterization-portuguese-patients-with-articulo-S0870255118302269
C Ruivo et al. ^22^	Myocardial deformation measures by cardiac magnetic resonance tissue tracking in myocarditis: relationship with systolic function and myocardial lesion

### Prevenção cardiovascular

A doença cardiovascular (DCV) continua a ser a principal causa de mortalidade a nível mundial. Apesar de atualmente dispormos de várias estratégias de tratamento da DCV, no “mundo real” o grau de controle dos fatores de risco cardiovascular (RCV) é inferior ao desejável. O estudo DISGEN-LIPID, ^2^ observacional e realizado em 24 centros em Portugal, teve por objetivo avaliar o grau de controle da dislipidemia, um dos principais fatores de RCV para o desenvolvimento de doença arterial coronária (DAC). Nesse estudo, observou-se que, apesar de a maioria dos doentes ser de alto ou muito alto RCV, mais de 50% daqueles em uso de hipolipemiantes não atingiram os valores-alvo recomendados de c-LDL, e que grandes porcentagens de doentes utilizavam estatinas de baixa intensidade ou em doses baixas. Curiosamente, outro estudo, analisando os dados do estudo DISGEN-LIPID, relatou uma disparidade significativa entre gêneros, com valores de perfil lipídico significativamente maiores nas mulheres do que nos homens. ^3^ Aquele estudo é relevante porque mostra a necessidade de se desenharem políticas de saúde pública que possam ultrapassar as barreiras atualmente existentes na implementação dos *guidelines* na prática clínica e ainda ultrapassar os problemas de subdosagem de estatina, a inércia terapêutica e a falta de adesão do doente ao tratamento.

Em 2019, a *Rev Port Cardiol* publicou outro importante estudo que ajuda a compreender o controle dos fatores de RCV na população portuguesa. O estudo PRECISE, ^4^ foi um estudo epidemiológico, transversal, que avaliou a prevalência dos vários fatores de RCV em 2848 doentes hipertensos seguidos nos cuidados primários de saúde. Os investigadores observaram que apenas 56% dos hipertensos tinham um bom controle da pressão arterial e que mais de 80% deles tinham três ou mais fatores de RCV. Esses dados mostram a importância de se fazer uma avaliação global do RCV no hipertenso e, mais uma vez, a necessidade urgente de implementar estratégias de prevenção que permitam melhorar o controle dos fatores de RCV em Portugal.

Ambos os estudos tiveram como primeiro autor o nosso colega Pedro Marques da Silva, médico ímpar, que deixa uma marca indelével na Medicina Cardiovascular. É com grande pesar que assistimos ao seu desaparecimento no início de 2020. Ficou mais pobre a Cardiologia em Portugal.

Em crianças e adolescentes também se observou dificuldade no controle dos fatores RCV. Em um estudo com 1254 crianças e adolescentes do sul do Brasil, 55% do sexo feminino, com idades entre 7 e 17 anos, foram analisadas as relações entre dislipidemia, fatores culturais e aptidão cardiorrespiratória (ACR). Os fatores culturais foram avaliados através de questionário autorreferido pelo escolar e os níveis de ACR, pelo teste de corrida/caminhada de 12 minutos, que consistiu em percorrer a maior distância possível em uma pista previamente demarcada durante 12 minutos. Os autores observaram prevalência de cerca de 42% de dislipidemia, que foi associada com o sexo feminino e baixos níveis de ACR. Na análise multivariada, dislipidemia foi associada com crianças e não com adolescentes, e com sobrepeso e obesidade. O deslocamento sedentário para a escola, grande tempo de permanência assistindo televisão, sexo feminino e presença de sobrepeso/obesidade também se associaram com os componentes isolados do perfil lipídico. Os autores ressaltaram a necessidade de intervenções precoces que promovam hábitos de vida saudáveis nas crianças. ^5^

O estudo ELSA-Brasil, ^6^ com 15.105 funcionários públicos, de 35 a 74 anos, vinculados a seis instituições de ensino e pesquisa das regiões sul, sudeste e nordeste do Brasil, avaliou a magnitude da associação entre a adiposidade abdominal, segundo diferentes indicadores diagnósticos [circunferência da cintura (CC), razão cintura quadril (RCQ), índice de conicidade, produto da acumulação lipídica (LAP), índice de adiposidade visceral (IAV)], e a espessura médio-intimal da carótida (EMIc), marcador de aterosclerose subclínica e preditor de infarto do miocárdio e acidente vascular cerebral (AVC). Os autores relataram, empregando regressão logística múltipla, que a adiposidade abdominal diagnosticada pela CC, mostrou importante associação com a EMIc em ambos os sexos (homens: OR = 1,47; IC95%: 1,22-1,77; mulheres: OR = 1,38; IC95%: 1,17-1,64). A adiposidade abdominal identificada pelos indicadores CC, RCQ, LAP e IAV entre as mulheres mostrou efeito de 0,02 mm sobre a EMIc (CC: 0,025, IC95%: 0,016-0,035; RCQ: 0,026, IC95%: 0,016-0,035; LAP: 0,024, IC95%: 0,014-0,034; IAV: 0,020, IC95%: 0,010-0,031). Os resultados observados reforçam a importância da adiposidade abdominal, representada pela CC, especialmente nos homens, como marcador simples da adiposidade abdominal associada com a aterosclerose subclínica.

A aterosclerose subclínica parece estar também associada com a inflamação sistêmica nos portadores de hipertensão arterial resistente (HAR). Procurou-se estabelecer um escore inflamatório (EI) em uma amostra de conveniência de 224 hipertensos, metade com HAR, empregando-se citocinas e adipocinas plasmáticas pró-inflamatórias e anti-inflamatórias, TNF-alfa, interleucinas (IL)-6, -8, -10, leptina e adiponectina. O EI correlacionou-se positivamente com índice de massa corporal (r = 0,40; p < 0,001), CC (r = 0,30; p < 0,001) e massa gorda avaliada por bioimpedância (r = 0,31; p < 0,001) em todos os indivíduos hipertensos. Poderá fornecer informação complementar na estratificação de RCV nos obesos com HAR. No entanto, precisará ser validado em outras populações para que seja recomendado para uso clínico, que ainda ficará limitado pelo alto custo da dosagem das citocinas e da adiponectina. ^7^

A obesidade, que foi associada com inflamação, também está associada com produção excessiva de espécies reativas de oxigênio. Com o objetivo de verificar os efeitos de oito semanas de treinamento resistido sobre os parâmetros de estresse oxidativo e inflamatórios em 24 camundongos *Swiss* com obesidade induzida por 26 semanas de dieta hiperlipídica, foi realizado teste de tolerância à insulina, monitoramento do peso corporal e marcadores de parâmetros de estresse oxidativo e inflamação no tecido cardíaco. Os resultados do estudo demonstraram controle do peso corporal apesar da ingesta excessiva de calorias, revertendo o dano em lipídios e a produção de espécies reativas de oxigênio, assim como modulando positivamente as principais citocinas responsáveis pela ativação do processo inflamatório. Dessa forma, o exercício resistido pode auxiliar no tratamento da obesidade, necessitando-se esclarecer como ele promove esses efeitos no tecido cardíaco. ^8^

Para 118 indivíduos, 77 hipertensos, foram ofertadas amostras aleatórias de pães com três concentrações de sal no tempo inicial e, após duas semanas, eles provaram os mesmos pães acrescidos de orégano, sendo aferida a pressão arterial e a excreção urinaria de sódio e potássio de 24 horas. Os idosos e jovens hipertensos preferiram e consumiram mais sal do que os normotensos, tendo o pão adicionado de tempero diminuído a preferência pelo sal nos jovens e nos idosos hipertensos. As variáveis que influenciaram significativamente a preferência por amostras de pão mais salgado foram: a presença de hipertensão, o sexo masculino e o consumo de álcool. Políticas públicas para diminuição da quantidade de sódio nos alimentos, como as realizadas em Portugal, possivelmente se associarão com melhor controle da hipertensão arterial e dos desfechos com ela relacionados, como o AVC, doença renal crônica e DAC. ^9^

### Doença coronariana crônica e síndromes coronarianas agudas

A introdução da angioplastia primária e da via verde coronariana permitiu uma redução marcada da mortalidade por síndrome coronariana aguda (SCA). ^10^ Contudo, é necessário continuar a melhorar a organização do sistema de angioplastia primária de forma a reduzir os tempos de atraso que são dependentes do sistema. A esse propósito, a *Rev Port Cardiol* publicou dois estudos relevantes. No primeiro, ^11^ foram avaliados 1222 doentes com infarto agudo do miocárdio com elevação do segmento ST (IAMcEST), mostrando-se que, em comparação com a orientação direta para o laboratório de hemodinâmica, a transferência inter-hospitalar dos doentes com IAMcEST aumentou de forma significativa o intervalo de tempo até a realização da angioplastia. No segundo estudo, ^12^ que faz parte da iniciativa *Stent* for Life, foram analisados os dados de 1340 doentes com IAMcEST admitidos em 18 hospitais portugueses com o objetivo de avaliar os indicadores de performance na população de alto risco, nomeadamente idosos, diabéticos e mulheres. Os investigadores mostraram que os idosos têm tempos de “atraso de doente” e de “atraso de sistema” muito maiores, independentemente do gênero e da presença de diabetes, demonstrando que esse grupo de doentes deve ser um alvo prioritário de novas estratégias de sensibilização da população.

No doente com infarto do miocárdio, existem vários escores para estratificação de risco, mas muitos deles são de difícil aplicabilidade no mundo real. Na edição de outubro da *Rev Port Cardiol* , Monteiro Pinto et al., ^13^ propõem um novo escore de risco de fácil utilização clínica, intitulado escore KAsH. Esse escore é calculado através de uma fórmula simples = (classe Killip x idade x frequência cardíaca) / pressão arterial sistólica. Num grupo de 1504 doentes consecutivos com infarto do miocárdio, esse novo escore mostrou ter melhor valor preditivo que os atualmente existentes, em especial o escore de GRACE. Sendo muito promissor, é agora necessária sua maior validação em outras coortes de doentes para sua posterior implementação na prática clínica. ^14^

A síndrome de Takotsubo é um dos diagnósticos diferenciais no doente com suspeita de SCA, sendo uma patologia que tem captado uma atenção crescente. ^15^ Este ano, foram publicados na *Rev Port Cardiol* os resultados de um estudo multicêntrico português, que avaliou as características de 234 doentes com esse diagnóstico. ^16^ Nesse artigo, mostrou-se que, em geral, essa síndrome tem um bom prognóstico a curto e a médio prazo (mortalidade intra-hospitalar de 2,2%), mas que a taxa de complicações intra-hospitalares (nomeadamente insuficiência cardíaca, fibrilação atrial, arritmias ventriculares e AVC) ainda é elevada (33%).

Com o objetivo de avaliar a expressão dos fatores transcricionais NF-κB e Nrf2 e o PPARβ/δ em pacientes com síndrome coronariana crônica (SCC), 35 pacientes com DAC (17 homens, com 62,4 ± 7,55 anos) e 12 sem DAC (5 homens, 63,50 ± 11,46 anos) foram estudados. As células mononucleares do sangue periférico (PBMCs) foram isoladas e processadas para a expressão de mRNA do Nrf2, NF-κB, NADPH: quinona oxidoredutase 1 (NQO1) e mRNAs do PPARβ/δ por meio de reação em cadeia da polimerase quantitativa em tempo real. Os autores observaram que o PPARβ/δ apresentou maior expressão nas PBMCs de pacientes com DAC comparados ao grupo controle, ao passo que não foram observadas diferenças nas expressões de mRNA do Nrf2 ou do NF-κB. Tais achados podem indicar possíveis terapias-alvo para pesquisas futuras na SCC. ^17^

Ainda em relação à SCC, foram estudados 5.526 pacientes obesos sem DAC conhecida, encaminhados para avaliação por CPM-SPECT entre janeiro de 2011 e dezembro de 2016. Os fatores associados à perfusão miocárdica anormal em pacientes obesos sem doença cardíaca isquêmica conhecida, após ajuste para as variáveis relevantes (análise multivariada), foram: idade (2% de aumento no risco para cada ano a mais de idade), diabetes mellitus (57% de aumento no risco em pacientes portadores dessa doença), angina típica (245% de aumento no risco em pacientes com angina típica quando comparados aos assintomáticos), utilização de estresse farmacológico durante o exame (61% de aumento no risco quando comparado ao estresse físico por meio de teste de esforço), menor intensidade do esforço físico avaliado em equivalente metabólico (MET: 10% de redução no risco para cada MET adicional realizado durante o teste de esforço) e fração de ejeção ventricular esquerda (FEVE) após estresse (1% de redução no risco para cada 1% a mais na FEVE). Esses dados corroboram a associação de obesidade e SCC. ^18^

### Arritmias cardíacas e dispositivos

A associação entre a fibrilação atrial e o risco de AVC é complexa e multifatorial. Ainda mais desafiante é a compreensão dos mecanismos envolvidos na ocorrência de AVC em doentes submetidos a hipocoagulação. Num estudo muito interessante publicado por Fernandes et al., ^19^ os investigadores estudaram 60 doentes consecutivos com fibrilação atrial não valvular, medicados cronicamente com anticoagulação oral e que, apesar disso, sofreram um AVC isquêmico. Na grande maioria desses doentes, a ocorrência do AVC parece ser explicada por subdosagem, má adesão terapêutica ou etiologia não cardioembólica e não por ineficácia desses fármacos, já que, em cerca de 90% dos doentes em uso de antagonistas da vitamina K, o INR na admissão era < 2 e, 43% daqueles tratados com anticoagulantes diretos estavam tomando subdosagem.

A implantação de cardiodesfibriladores implantáveis (CDI) e de dispositivos de ressincronização ventricular (CRT) permite a redução do risco de morte e hospitalização e uma melhoria da qualidade de vida nos doentes com insuficiência cardíaca com FEVE reduzida. Bonhorst et al. publicaram na *Rev Port Cardiol* os resultados do estudo Síncrone, ^20^ que é um registro observacional, multicêntrico e prospetivo realizado em 16 centros portugueses e que incluiu 486 doentes com diagnóstico de insuficiência cardíaca, FEVE < 35% e indicação para implantação de CDI ou CRT. Nesse estudo, a maioria dos doentes tratados com dispositivos tinha uma recomendação de classe I dos *guidelines* , sendo as taxas de mortalidade com 1 ano baixas (apenas 3,6%), assim como o número de reinternações (11%). Esse estudo ajuda a compreender a realidade do tratamento com dispositivos para insuficiência cardíaca em Portugal, mostrando ainda a necessidade de se melhorar o tratamento farmacológico desses doentes já que as taxas de utilização de medicação foram sub-ótimas (76% inibidor da enzima conversora de angiotensina/antagonista do receptor de aldosterona; 77% bloqueadores beta-adrenérgicos; 34% antagonista da aldosterona).

### Insuficiência cardíaca e miocardiopatias

Nos doentes com miocardiopatia hipertrófica (MH), as diretrizes europeias recomendam a avaliação do risco de morte súbita de acordo com o escore ESC-SCD. ^21^ Contudo, têm surgido várias críticas a esse escore, sendo necessário criar novas formas de estratificação do risco nesses doentes. Ruivo C et al., ^22^ publicaram na *Rev Port Cardiol* um estudo interessante baseado nos dados do registro nacional de MH, que inclui 1022 doentes com essa patologia. Após identificação dos principais determinantes de risco de morte súbita, os investigadores construíram um novo modelo de risco designado escore SHIFT, que inclui quatro variáveis: síncope inexplicada, sinais de insuficiência cardíaca, espessura do septo ≥ 19 mm e presença de complexo QRS fragmentado. Nessa população de doentes, o escore SHIFT, que integra parâmetros clínicos, eletrocardiográficos e ecocardiográficos relativamente simples de se avaliar, teve melhor valor preditivo (C-index 0.81) que o escore ESC-SCD. Por isso, no futuro, esse novo escore poderá desempenhar um papel importante para ajudar a selecionar os doentes com MH e indicação para implantação de CDI em prevenção primária.

Na área da miocardiopatia dilatada, a *Rev Port Cardiol* publicou em 2019 um estudo multicêntrico que pretendeu fazer uma caracterização molecular e genética de 107 doentes com cardiomiopatia dilatada. ^23^ Os investigadores observaram uma grande complexidade e diversidade genética nesses doentes com identificação de 31 variantes genéticas raras em oito genes diferentes, que envolvem sobretudo genes sarcoméricos (MYBPC3, TNNT2 e LMNA). Esse estudo reforça a importância das novas técnicas de análise genômica, sobretudo por técnicas de *next-generation sequencing* , para ajudar a compreender a etiologia dessa doença.

Nos últimos anos, a ressonância cardíaca tem desempenhado um papel crescente na avaliação dos doentes com miocardite, sendo atualmente o exame não invasivo de eleição para o diagnóstico dessa patologia. ^24^ Num estudo publicado na *Rev Port Cardiol* em 2019, ^25^ os investigadores avaliaram o papel da quantificação da deformação miocárdica por *tissue tracking* como uma medida objetiva de quantificação de função em 78 doentes com miocardite, tendo encontrado correlações significativas de todos os parâmetros de deformação ( *strain, strain rate* , velocidade e deslocamento) com a FEVE, com as alterações segmentares e a quantidade de realce tardio. Agora o desafio será compreender de que forma esses resultados podem influenciar a abordagem clínica dos doentes com miocardite. ^26^

A avaliação do risco de cardiotoxicidade dos quimioterápicos da classe das antraciclinas e dos anticorpos monoclonais humanizados é um desafio clínico, que estimula a busca por preditores de alteração contrátil segmentar ventricular esquerda (ACSVE) que sejam de fácil realização e que permitam reavaliações ao longo do tratamento. Barros et al., ^27^ estudando 112 pacientes, média de idade de 51,3 ± 12,9 anos, com câncer de mama e tratamento com doxorrubicina e/ou trastuzumabe, realizaram estudo ecocardiográfico para avaliar cardiotoxicidade, que foi definida por decréscimo de 10% na FEVE. A presença de cardiotoxicidade foi observada em 18 (16,1%) pacientes. Na análise multivariada, ACSVE (OR = 6,25 [IC 95%: 1,03; 37,95], p < 0,05), diâmetro sistólico do ventrículo esquerdo (OR = 1,34 [IC 95%: 1,01; 1,79], p < 0,05) e *strain* longitudinal global pela técnica de *speckle tracking* (OR = 1,48 [IC 95%: 1,02; 2,12], p < 0,05) foram preditores significativos e independentes de cardiotoxicidade. Os autores concluíram que a ACSVE é preditor independente de cardiotoxicidade e poderá ser útil para detecção precoce da disfunção miocárdica. ^27^

### Cirurgia Cardíaca

Em meta-análise de quatro estudos controlados e randomizados que buscava determinar os desfechos clínicos de 1528 pacientes portadores de próteses valvares mecânicas e biológicas, antigas e contemporâneas, seguidos entre 2 e 20 anos, não foram observadas diferenças para os desfechos morte (risco relativo, RR = 1,07; IC95%: 0,99-1,15), embolia arterial sistêmica (RR = 0,93; IC95%: 0,66-1,31) e endocardite infecciosa (RR = 1,21; IC95%: 0,78-1,88) entre os portadores de próteses mecânicas e biológicas. Por outro lado, o risco de sangramento foi um terço menor (RR = 0,64; IC95%: 0,52-0,78) e o número de reoperações (RR = 3,60; IC95%: 2,44-5,32) foi três vezes maior nos pacientes com próteses biológicas. Três estudos incluíram próteses de antiga geração, que apresentaram resultados similares às de nova geração. Os autores salientam a ausência de estudos com as novas gerações de próteses, exortando a realização de estudos que comparem próteses biológicas e mecânicas contemporâneas. ^28^

### Cardiopatias congênitas

A gravidez em portadoras de cardiopatia congênita complexa (CCC) tornou-se uma realidade e o manejo materno-fetal é um desafio clínico atual. Avila et al., ^29^ estudaram 435 gestantes portadoras de CCC, por um período de 10 anos, que foram incluídas no Registro do Instituto do Coração (Registro-InCor). Selecionaram 42 gestações em 40 mulheres com CCC (24,5 ± 3,4 anos) que foram desaconselhadas a engravidar. As CCC listadas foram transposição das grandes artérias, atresia pulmonar, atresia tricúspide, ventrículo único, dupla via de saída de ventrículo direito, dupla via de entrada de ventrículo esquerdo, que foram tratadas com cirurgias Rastelli, Fontan, Jatene, Senning, Mustard e outros procedimentos combinados, como tunelização, Blalock Taussig e Glenn. Das 40 mulheres com CCC, 8 não foram submetidas a cirurgia e cerca de 48% apresentaram hipoxemia. Apesar do acompanhamento individualizado e frequente, com hospitalização a partir de 28 semanas, a maior parte das gestações, cerca de 60%, apresentou complicações maternas ou fetais. Foram relatadas complicações maternas em 31% das gestações, incluindo 2 mortes causadas por hemorragia pós-parto e pré-eclâmpsia grave, e 7 perdas fetais, 17 bebês prematuros e 2 recém-nascidos com cardiopatia congênita. Os autores reforçaram as orientações vigentes de que, apesar da melhoria no prognóstico das CCC e da necessidade de se respeitar a autonomia da intenção à concepção, as complicações maternas e fetais desaconselham a gravidez, especialmente nas pacientes que apresentam hipoxemia.

## Conclusões Finais

Esta revisão dos melhores do ano, sob o ponto de vista dos editores dos *Arq Bras Cardiol* e da *Rev Port Cardiol* , é uma pequena amostra de tudo que essas publicações científicas têm a oferecer em termos de atualização e divulgação de inovações para os seus leitores. Ela coloca em evidência a relevância da ciência em língua portuguesa. Procuramos trazer para os leitores a melhor informação no melhor formato, sucinto, preciso e eficiente.

A ciência só progride quando o conhecimento é compartilhado. O papel dos *Arq Bras Cardiol* e da *Rev Port Cardiol* é publicar, circular e divulgar cada vez mais a ciência por elas veiculada e contribuir para o progresso científico global. E por que não o fazer também de forma elegante e eficiente na nossa amada língua-mãe, com o sotaque que mais lhe agradar? Esperamos que todos tenham apreciado esse resumo do melhor do ano de 2019 e os aguardamos ano que vem com o melhor de 2020. Convidamos também os nossos leitores, associados das nossas sociedades de Cardiologia, cardiologistas, médicos e cientistas em geral a se manterem conectados com nossas publicações científicas de forma constante, pelas vias digitais tradicionais (webpage), redes sociais (Facebook, Twitter e LinkedIn) e aplicativos de smartphones. Boa leitura a todos!
