# Supplementary material for: Os Top 10 Artigos Originais Publicados nos Arquivos Brasileiros de Cardiologia e na Revista Portuguesa de Cardiologia em 2019
Source: Arq Bras Cardiol. 2020 Mar 20;114(3):564–70. [Article in Portuguese] doi: 10.36660/abc.20200176 (PMC7792718; doi:10.36660/abc.20200176)
Supplement: Translation

# Translation: The Top 10 Original Articles Published in the *Arquivos Brasileiros de Cardiologia* and in the *Revista Portuguesa de Cardiologia* in 2019

**Authors:** Gláucia Maria Moraes de Oliveira, Ricardo Fontes-Carvalho, Lino Gonçalves, Nuno Cardim, Carlos Eduardo Rochitte

**Affiliations:** 1 Brazil Faculdade de Medicina – Universidade Federal do Rio de Janeiro, Rio de Janeiro, RJ – Brazil; 2 Brazil Instituto do Coração Edson Saad – Universidade Federal do Rio de Janeiro, Rio de Janeiro, RJ – Brazil; 3 Portugal Departamento de Cardiologia – Centro Hospitalar de Vila Nova de Gaia, Vila Nova de Gaia – Portugal; 4 Portugal Departamento de Cirurgia e Fisiologia – Faculdade de Medicina – Universidade do Porto, Porto – Portugal; 5 Portugal Departamento de Cardiologia – Centro Hospitalar e Universitário de Coimbra, Coimbra – Portugal; 6 Portugal Faculdade de Medicina – Universidade de Coimbra, Coimbra – Portugal; 7 Portugal Departamento de Cardiologia do Hospital da Luz, Lisboa - Portugal; 8 Portugal Faculdade de Ciências Médicas da Universidade Nova de Lisboa, Lisboa - Portugal; 9 Brazil Instituto do Coração (InCor) – Faculdade de Medicina da Universidade de São Paulo, São Paulo, SP – Brazil; 10 Brazil Hospital do Coração (HCOR), São Paulo, SP – Brazil

**Keywords:** Cardiovascular Diseases, Periodicals as Topic, Portals for Scientific Journals, Journal Impact Factor, Periodical, System for Evaluation of Publications

## Introduction

For centuries, Portugal and Brazil have shared a tradition of cooperation, which applies to medicine. In cardiology, for example, their national scientific societies – the Brazilian Society of Cardiology and the Portuguese Society of Cardiology – have a long history of collaboration, which extends to their journals.

The *Arquivos Brasileiros de Cardiologia* ( *Arq Bras Cardiol* ), the official scientific publication of the Brazilian Society of Cardiology, with an impact factor of 1.679 in 2018 (JCR), is the most influential cardiology journal in Brazil and Latin America. This can be exemplified by the increasing number of submissions to the *Arq Bras Cardiol* (650 in 2017, 771 in 2018, and 734 in 2019), and by its h5 index of 31 and h5 median of 39, with acceptance rate lower than 20%.

The *Revista Portuguesa de Cardiologia* ( *Rev Port Cardiol* ) is the official journal of the Portuguese Society of Cardiology, an institutional member of the European Society of Cardiology. It has been continuously published since 1982, has global impact, being indexed in PubMed, Elsevier, ScienceDirect and SCOPUS, with an impact factor of 0.79 in 2018.

In that year, for the first time, both journals got together to issue a review of the most relevant original papers published in both journals in 2018.^1^Because of the great success of that initiative, the editorial bodies of those two journals decided to cooperate again to select their best 2019 publications. The articles of the *Arq Bras Cardiol* listed here were those selected for the Brazilian Society of Cardiology Publication Award. It is worth noting that Nuno Cardim, author of the best article published in the *Rev Port Cardiol* in 2019, attended the award ceremony of the 74^th^Brazilian Congress of Cardiology, in the city of Porto Alegre, in 2019. We aim to strengthen the cultural ties of the major cardiology journals published in Portuguese, which represent the best publications directed to a growing population of around 250 million people worldwide.

Given the overall high quality of the articles published, this selection was a hard, possibly imperfect task, which allowed us to highlight several relevant papers in cardiology. Tables 1 and 2 list the top ten articles published in each journal in 2019.

**Table 1:** – List of the top ten articles published in the Arquivos Brasileiros de Cardiologia in 2019

Author	Title of the article and link
Faria AP et al.^7^	Proposta de um Escore Inflamatório de Citocinas e Adipocinas Plasmáticas Associado à Hipertensão Resistente, mas Dependente dos Parâmetros de Obesidade http://www.scielo.br/scielo.php?script=sci_arttext&pid=S0066-782X2019000400383&lng=pt&nrm=iso&tlng=pt
Dippe Jr. et al.^18^	Estudo de Perfusão Miocárdica em Obesos sem Doença Cardíaca Isquêmica Conhecida http://www.scielo.br/scielo.php?script=sci_arttext&pid=S0066-782X2019000200121&lng=en&nrm=iso&tlng=pt
Reuter CP et al.^5^	Relação entre Dislipidemia, Fatores Culturais e Aptidão Cardiorrespiratória em Escolares http://www.scielo.br/scielo.php?script=sci_arttext&pid=S0066-782X2019000600729&lng=en&nrm=iso&tlng=pt
Villela PTM et al.^9^	A Preferência ao Sal está Relacionada à Hipertensão e não ao Envelhecimento http://www.scielo.br/scielo.php?pid=S0066-782X2019005015104&script=sci_arttext&tlng=pt
Barros MVL et al.^27^	Alteração Contrátil Segmentar Ventricular Esquerda é Preditor Independente de Cardiotoxicidade em Pacientes com Câncer de Mama em Tratamento Quimioterápico http://www.scielo.br/scielo.php?script=sci_arttext&pid=S0066-782X2019000100050&lng=pt&nrm=iso&tlng=pt
Kiyose AT et al.^28^	Comparação de Próteses Biológicas e Mecânicas para Cirurgia de Válvula Cardíaca: Revisão Sistemática de Estudos Controlados Randomizados http://www.scielo.br/scielo.php?script=sci_arttext&pid=S0066-782X2019000300292&lng=en&nrm=iso&tlng=pt
Eickemberg M et al.^6^	Indicadores de Adiposidade Abdominal e Espessura Médio-Intimal de Carótidas: Resultados do Estudo Longitudinal de Saúde do Adulto - ELSA-Brasil http://www.scielo.br/scielo.php?script=sci_arttext&pid=S0066-782X2019000300220&lng=en&nrm=iso&tlng=pt
Effting PS et al.^8^	Exercício Resistido Modula Parâmetros de Estresse Oxidativo e Conteúdo de TNF-α no Coração de Camundongos com Obesidade Induzida por Dieta http://www.scielo.br/scielo.php?script=sci_arttext&pid=S0066-782X2019000500545&lng=es&nrm=iso&tlng=pt
Avila WS et al.^29^	Gravidez em portadoras de cardiopatias congênitas complexas. Um constante desafio http://www.scielo.br/scielo.php?pid=S0066-782X2019005019101&script=sci_arttext&tlng=pt
Barbosa JE et al.^17^	Perfil da Expressão do mRNA do Nrf2, NF-κB e PPARβ/δ em Pacientes com Doença Arterial Coronariana http://www.scielo.br/scielo.php?script=sci_arttext&pid=S0066-782X2019005001205&lng=en&nrm=iso&tlng=pt

**Table 2:** – List of the top ten articles published in the Revista Portuguesa de Cardiologia in 2019.

Author	Title of the article
P Marques Silva et al.^2^	Suboptimal lipid levels in clinical practice among Portuguese adults with dyslipidemia under lipid-lowering therapy: Data from the DISGEN-LIPID study
P Marques Silva et al.^4^	Prevalência de fatores de risco cardiovascular e outras comorbilidades em doentes com hipertensão arterial assistidos nos Cuidados de Saúde Primários: estudo PRECISE
R Calé et al.^12^	Time to reperfusion in high-risk subgroup patients with myocardial infarction undergoing primary percutaneous coronary intervention
J Pinto Monteiro et al.^13^	KAsH: Uma nova ferramenta para previsão de mortalidade hospitalar em doentes com Enfarte Agudo do Miocárdio
D Bento et al.^16^	Short and medium-term prognosis of Takotsubo syndrome in a Portuguese Population
D Bonhorst et al.^20^	Implantação de dispositivos de ressincronização e/ou desfibrilhação em doentes com insuficiência cardíaca: dados da vida real – o Estudo Síncrone
L Fernandes et al.^19^	Acidente vascular cerebral isquémico em doentes previamente anticoagulados por fibrilhação auricular não valvular: por que acontece?
C Ruivo et al.^25^	The SHIFT model combines clinical, electrocardiographic and echocardiographic parameters to predict Sudden Cardiac Death in Hypertrophic Cardiomyopathy
A Sousa et al.^23^	Molecular characterization of Portuguese patients with dilated cardiomyopathy
C Ruivo et al.^22^	Myocardial deformation measures by cardiac magnetic resonance tissue tracking in myocarditis: relationship with systolic function and myocardial lesion

### Cardiovascular prevention

Cardiovascular disease (CVD) remains the leading cause of mortality worldwide. Although several strategies to treat CVD are currently available, the control of cardiovascular risk (CVR) factors remains below the desired degree. The DISGEN-LIPID^2^study, an observational study conducted in 24 centers in Portugal, has aimed at assessing the degree of control of dyslipidemia, one of the major CVR factors for the development of coronary artery disease (CAD). That study has shown that, although most patients were at high or very high CVR, more than 50% of those on lipid-lowering therapy did not achieve the recommended target levels for LDL-C, a large proportion being on low-intensity statins or low-dose therapy. Another study, assessing data from the DISGEN-LIPID study, has shown a significant disparity between genders, with lipid profile values significantly higher in women than in men.^3^That relevant study evidences the need for public health policies that can overcome both the current obstacles to implementing the guidelines in clinical practice and the problems of low statin dose, therapeutic inertia and lack of patient’s adherence to treatment.

In 2019, the *Rev Port Cardiol* published the PRECISE study^4^to help understand the control of CVR factors in the Portuguese population. That epidemiological, cross-sectional study has assessed the prevalence of several CVR factors in 2848 hypertensive patients followed up in primary health care centers. The study has shown that only 56% of those patients had good blood pressure control and more than 80% of them had three or more concomitant CVR factors. These data show the importance of the overall assessment of CVR in hypertensive patients, and, once again, the urgent need to implement preventive strategies to improve the control of CVR factors in Portugal.

The first author of both studies was our colleague Pedro Marques da Silva, a remarkable physician, who left an indelible mark on Cardiovascular Medicine. His passing in early 2020 brought us great sorrow. This was a tremendous loss for cardiology in Portugal.

Difficulty controlling CVR factors in children and adolescents has also been observed. A study with 1254 children and adolescents from southern Brazil (female sex, 55%; age range: 7-17 years) has assessed the relationship between dyslipidemia, cultural factors and cardiorespiratory fitness (CRF). The cultural factors were assessed by use of a self-reporting questionnaire, and the CRF levels, by use of the 12-minute walk/run test, which consisted in covering the longest possible distance on a previously established track for 12 minutes. The authors have reported a dyslipidemia prevalence of 42%, which was associated with female sex and low CRF levels. On multivariate analysis, dyslipidemia was associated with children, but not with adolescents, as well as with overweight and obesity. In addition, sedentary commuting to and from school, too much time spent watching TV, female sex, and overweight/obesity were associated with isolated components of the lipid profile. The authors have emphasized the need for early interventions that promote healthy life habits among children.^5^

The ELSA-Brazil study,^6^assessing 15105 public servers (age range: 35-74 years) from six teaching and research institutions of the Brazilian southern, southeastern and northeastern regions, has assessed the magnitude of the association between abdominal adiposity, defined based on different diagnostic indicators [waist circumference (WC), waist-to-hip ratio (WHR), conicity index , lipid accumulation product (LAP), visceral adiposity index (VAI)], and carotid intima-media thickness (cIMT), a marker of subclinical atherosclerosis and predictor of myocardial infarction and stroke. By using multiple logistic regression, the authors have shown an important association of abdominal adiposity, diagnosed by use of WC, with cIMT in both sexes (men: OR = 1.47; 95% CI: 1.22-1.77; women: OR = 1.38; 95% CI: 1.17-1.64). Abdominal adiposity, identified by the indicators WC, WHR, LAP and VAI, in women showed a 0.02-mm effect on cIMT (WC: 0.025, 95% CI: 0.016-0.035; WHR: 0.026, 95% CI: 0.016-0.035; LAP: 0.024, 95% CI: 0.014-0.034; VAI: 0.020, 95% CI: 0.010-0.031). The results observed reinforce the importance of abdominal adiposity, represented by WC, especially in men, as a simple marker of abdominal adiposity associated with subclinical atherosclerosis.

In addition, subclinical atherosclerosis seems to be associated with systemic inflammation in patients with resistant arterial hypertension (RAH). In a convenience sample of 224 hypertensive patients, half of them with RAH, an inflammatory score (IS) was established, comprising the measurement of plasma pro-inflammatory and anti-inflammatory cytokines and adipokines, TNF-alpha, interleukins (IL)-6, -8, -10, leptin and adiponectin. The IS correlated positively with body mass index (r = 0.40; p < 0.001), WC (r = 0.30; p < 0.001) and fat mass, assessed by use of bioimpedance (r = 0.31; p < 0.001) in all hypertensive individuals. It may provide complementary information on CVR stratification in obese individuals with RAH. However, it requires validation in other populations to be recommended for clinical use, which is limited by the high cost of measuring cytokines and adiponectin.^7^

Obesity, which was associated with inflammation, is also associated with the excessive production of reactive oxygen species. Aiming to assess the effects of an 8-week resisted training on oxidative stress and inflammatory parameters in 24 Swiss mice with obesity induced by a 26-week lipid-rich diet, insulin tolerance testing was performed and body weight was monitored, as were oxidative stress markers and inflammatory parameters on cardiac tissue. The results of the study have shown body weight control despite the excessive calory intake, reversing the lipid damage and the production of reactive oxygen species, and positively modulating the major cytokines responsible for activating the inflammatory process. Thus, resisted exercise can aid the treatment of obesity, but how it promotes those effects on cardiac tissue requires clarification.^8^

In another study, 118 individuals, 77 of whom were hypertensive, received random samples of bread with three different salt contents at the beginning of the study. After two weeks, the individuals received the same breads, then added with oregano, and had their arterial blood pressure and 24-hour urine sodium and potassium excretion measured. The hypertensive elderly and young individuals preferred and consumed more salt than the normotensive individuals, and the bread added with oregano decreased the preference for salt in hypertensive elderly and young individuals. The variables that significantly influenced the preference for saltier bread samples were: hypertension, male sex, and alcohol consumption. Public policies to reduce the sodium content of meals, such as those implemented in Portugal, might associate with better control of arterial hypertension and its outcomes, such as stroke, chronic kidney disease and CAD.^9^

### Chronic coronary artery disease and acute coronary syndromes

The introduction of direct access to primary angioplasty has allowed for a significant reduction in mortality due to acute coronary syndrome (ACS).^10^However, the organization of the primary angioplasty system requires continuous improvement to reduce system-dependent delay times. *The Rev Port Cardiol* has published two relevant studies about that. The first study,^11^assessing 1222 patients with ST-segment elevation myocardial infarction (STEMI), has shown that, as compared to patients admitted directly to a catheterization laboratory, the inter-hospital transfer of patients with STEMI significantly increased ischemic time. The second study,^12^part of the Stent for Life initiative, has assessed data on 1340 patients with STEMI admitted to 18 Portuguese hospitals, aiming at evaluating the performance indicators in the high-risk population, namely elderly, diabetic and female patients. The authors have reported that the elderly have longer patient and system delays, regardless of gender and presence of diabetes, suggesting that the elderly subgroup should be the target of new sensitization strategies.

There are several scores for the risk stratification of patients with myocardial infarction, many of which are difficult to use. On the October edition of the *Rev Port Cardiol* , Monteiro Pinto et al.^13^have proposed a new simple to use clinical score, the KAsH score, which is calculated according to the following formula: KAsH = (Killip class x age x heart rate)/systolic blood pressure. In 1504 consecutively admitted patients with myocardial infarction, the new score has shown a better predictive value than the existing scores, specially the GRACE score. Although promising, the KAsH score requires better validation in other cohorts of patients to be then implemented in clinical practice.^14^

The Takotsubo syndrome is a differential diagnosis for patients suspected of having ACS, and has gained increasing attention.^15^This year, the *Rev Port Cardiol* published the results of a Portuguese multicenter study that has assessed the characteristics of 234 patients diagnosed with Takotsubo syndrome.^16^That study has shown that the Takotsubo syndrome has a good short- and medium-term prognosis (in-hospital mortality of 2.2%), but the rate of in-hospital complications (namely heart failure, atrial fibrillation, ventricular arrhythmias and stroke) is high (33%).

Aiming at assessing the expression of the transcriptional factors NF-κB and Nrf2 and PPARβ/δ in chronic coronary syndrome (CCS), 35 patients with CAD (17 men; mean age, 62.4 ± 7.55 years) and 12 patients without CAD (5 men; mean age, 63.50 ± 11.46 years) were studied. Peripheral blood mononuclear cells (PBMC) were isolated and processed for the mRNA expression of Nrf2, NF-κB, NADPH:quinone oxidoreductase 1 (NQO1) and PPARβ/δ by use of real-time quantitative polymerase chain reaction. The authors have reported a higher mRNA expression of PPARβ/δ in the PBMC of patients with CAD as compared to that of the control group, while the mRNA expressions of Nrf2 and NF-κB did not differ. Such findings might indicate possible target-therapies for future research in CCS.^17^

Another study on CCS has evaluated 5526 obese patients without known CAD referred for CPM-SPECT assessment between January 2011 and December 2016. The factors associated with abnormal myocardial perfusion in obese patients without known ischemic heart disease after adjusting for the relevant variables (multivariate analysis) were: age (2% risk increase for each year of age); diabetes mellitus (57% risk increase); typical angina (245% risk increase in patients with typical angina as compared to asymptomatic patients); need for pharmacologic stress during testing (61% risk increase as compared to physical stress by use of exercise testing); less physical effort evaluated in metabolic equivalents (METs - 10% risk reduction for each additional MET during exercise testing); and left ventricular ejection fraction (LVEF) after stress (1% risk reduction for each 1% addition in LVEF). Such data support the association of obesity and CCS.^18^

### Cardiac arrhythmias and devices

The association between atrial fibrillation and the risk of stroke is complex and multifactorial. Even more challenging is understanding the mechanisms involved in the occurrence of stroke in patients undergoing anticoagulation. In a very interesting study, Fernandes et al.^19^have assessed 60 consecutive patients with nonvalvular atrial fibrillation, chronically medicated with an oral anticoagulant and admitted due to ischemic stroke. For most of those patients, stroke occurrence despite anticoagulation appears to be explained by subtherapeutic dosage, poor treatment adherence or non-cardioembolic etiology, and not by inefficacy of the anticoagulants, because 90% of the patients on vitamin K antagonists had an admission INR < 2, and subtherapeutic prescriptions were found in 43% of those on novel oral anticoagulants.

Implantable cardioverter defibrillator (ICD) and cardiac resynchronization therapy (CRT) reduce the risk of death and hospitalization and promote an improvement in the quality of life of patients with heart failure and reduced LVEF. Bonhorst et al. have published in the *Rev Port Cardiol* the results of the Síncrone study,^20^an observational, prospective, multicenter registry conducted in 16 centers in Portugal that included 486 patients with a diagnosis of heart failure, LVEF < 35% and indication for ICD or CRT devices. In that study, most patients treated with devices had a class I recommendation of the guidelines, the overall mortality at one year being low (3.6%), as was the number of hospitalizations (11%). That study helps understand the reality of the treatment with devices for heart failure in Portugal. In addition, it evidences the need to improve the pharmacological treatment of those patients, because the drug use rates were suboptimal (76% angiotensin-converting-enzyme inhibitor/aldosterone receptor antagonist; 77% beta-adrenergic blockers; 34% aldosterone antagonist).

### Heart failure and cardiomyopathies

For patients with hypertrophic cardiomyopathy (HCM), the European guidelines recommend assessing the risk of sudden death according to the ESC-SCD score.^21^However, that score has come under criticism, and new risk stratification models are required for those patients. Ruivo C et al.^22^have published in the *Rev Port Cardiol* a study based on data from the Portuguese National Registry of HCM, which includes 1022 patients with HCM. After identifying the major determinants of the risk of sudden death, the authors have built a new risk model, the SHIFT score, which includes four variables: unexplained syncope; signs of heart failure; septal thickness ≥ 19 mm; and fragmented QRS complex. In that population of patients, the SHIFT score, which includes relatively simple clinical, electrocardiographic and echocardiographic parameters, showed a better predictive value (C-index, 0.81) than the ESC-SCD score. Thus, that new score might play an important role in selecting patients with HCM with indication for ICD in primary prevention.

Regarding dilated cardiomyopathy, the *Rev Port Cardiol* published in 2019 a multicenter study aimed at providing the molecular and genetic characterization of 107 patients with dilated cardiomyopathy.^23^The authors have reported large genetic complexity and diversity in those patients, having identified 31 rare variants in eight different genes, mainly involving sarcomeric genes (MYBPC3, TNNT2 and LMNA). That study emphasizes the importance of the new genomic analysis techniques, mainly next-generation sequencing techniques, to better understand the etiology of dilated cardiomyopathy.

Cardiovascular magnetic resonance has played an increasing role in the assessment of patients with myocarditis, being currently the non-invasive test of choice to diagnose that pathology.^24^A study^25^published in the *Rev Port Cardiol* in 2019 has assessed the role of quantifying myocardial deformation by using tissue tracking as an objective measure of myocardial function quantification in 78 patients with myocarditis. Significant correlations were found between all deformation parameters (strain, strain rate, velocity and displacement) and LVEF, regional wall motion abnormalities, and the extent of late gadolinium enhancement. The challenge now is to understand how those results can influence the clinical approach of patients with myocarditis.^26^

Assessing the risk of cardiotoxicity of anthracycline chemotherapy and humanized monoclonal antibodies is a clinical challenge that stimulates the search for predictors of left ventricular regional wall motion abnormalities (LVRWMA) that are easy to use and allow reassessment throughout treatment. Barros et al.,^27^evaluating 112 patients (mean age, 51.3 ± 12.9 years) with breast cancer and treated with doxorubicin and/or trastuzumab, have carried out an echocardiographic study to assess cardiotoxicity, which was defined as a 10% decrease in LVEF. Cardiotoxicity was observed in 18 (16.1%) patients. On multivariate analysis, LVRWMA (OR = 6.25 [95% CI: 1.03; 37.95], p < 0.05), left ventricular systolic diameter (OR = 1.34 [95% CI: 1.01; 1.79], p < 0.05) and global longitudinal strain by speckle tracking (OR = 1.48 [95% CI: 1.02; 2.12], p < 0.05) were significant and independent predictors of cardiotoxicity. Those authors have concluded that LVRWMA is an independent predictor of cardiotoxicity and can be useful in the early detection of myocardial dysfunction.^27^

### Heart Surgery

A meta-analysis of four controlled randomized studies, aimed at determining the clinical outcomes of 1528 patients with old-generation and contemporary mechanical and biological valvular prostheses, followed up for 2-20 years, showed no difference between patients with mechanical and biological valvular prostheses regarding the outcomes death (relative risk, RR = 1.07; 95% CI: 0.99-1.15), systemic arterial embolism (RR = 0.93; 95% CI: 0.66-1.31), and infective endocarditis (RR = 1.21; 95% CI: 0.78-1.88). However, the risk of bleeding was one-third lower (RR = 0.64; 95% CI: 0.52-0.78) and the number of reoperations (RR = 3.60; 95% CI: 2.44-5.32) was three times higher in patients with biological valvular prostheses. Three studies had included old-generation valvular prostheses with results similar to those of the new generation ones. The authors highlight the lack of studies on the new generation valvular prostheses, emphasizing the need to compare contemporary mechanical and biological valvular prostheses.^28^

### Congenital heart diseases

Pregnancy in patients with complex congenital heart disease (CCHD) has become a reality, maternal-fetal management being a current clinical challenge. Avila et al. have studied, for 10 years, 435 pregnant women with CCHD, included in the registry of the Instituto do Coração (Registro-InCor). They have selected 42 pregnancies in 40 women with CCHD (24.5 ± 3.4 years), who had been advised not to get pregnant. The CCHD listed were as follows: transposition of the great arteries, pulmonary atresia, tricuspid atresia, single ventricle, double right ventricular outflow tract, and double left ventricular inlet. Those CCHD had been treated with the Rastelli, Fontan, Jatene, Senning and Mustard surgeries, and other procedures combined, such as tunneling, Blalock Taussig and Glenn. Of the 40 women with CCHD, 8 had not undergone surgery and 48% were hypoxemic. Despite the individualized and frequent follow-up, with hospitalization from the 28th week onward, most pregnancies (60%) had maternal or fetal complications, as follows: maternal complications reported in 31% of the pregnancies, including two deaths caused by post-partum hemorrhage and severe pre-eclampsia; 7 fetal losses; 17 premature babies; and 2 newborns with congenital heart disease. Despite the improvement in the prognosis of CCHD and the need to respect a woman’s intention to conceive, the authors highlight the current recommendations that maternal and fetal complications advise against pregnancy, especially in hypoxemic patients.

## Final Conclusions

From the perspective of the editors of the *Arq Bras Cardiol* and the *Rev Port Cardiol* , this review of the best articles of the year is a small sample of what such scientific publications have to offer regarding updating and spreading of innovations to their readers. This review evidences the relevance of science in the Portuguese language. We aimed to provide the readers with the best information, in a brief, precise and efficient way.

Science only moves forward when knowledge is shared. The role of the *Arq Bras Cardiol* and the *Rev Port Cardiol* is to publish, circulate and disseminate science, as well as to contribute to the global scientific progress. And why should we not do that elegantly and efficiently in our beloved mother language, with the accent that pleases us most? We hope everybody enjoys this review of the 2019 best articles and we are looking forward to the 2020 best ones. In addition, we invite our readers, members of our societies of cardiology, cardiologists, physicians and scientists in general to remain constantly connected to our scientific publications by using the traditional digital way (webpage), social media (Facebook, Twitter and LinkedIn), and smartphone apps. Enjoy the reading!
